# Diagnostic performance of short noncontrast biparametric 3-T MRI for tonsillar infections: comparison with a full protocol including contrast-enhanced sequences

**DOI:** 10.1186/s41747-023-00379-0

**Published:** 2023-10-24

**Authors:** Jari-Pekka Vierula, Janne Nurminen, Ville Jussila, Mikko Nyman, Jaakko Heikkinen, Bernd Pape, Kaarlo Sorvettula, Kimmo Mattila, Jussi Hirvonen

**Affiliations:** 1 Department of Radiology, Turku University Hospital, Turku, Finland; 2 Department of Biostatistics, Turku University Hospital, Turku, Finland; 3 School of Technology and Innovations, University of Vaasa, Vaasa, Finland; 4 Medical Imaging Center, Department of Radiology, Tampere University and Tampere University Hospital, Tampere, Finland

**Keywords:** Emergency medicine, Magnetic resonance imaging, Observer variation, Peritonsillar abscess, Tonsillitis

## Abstract

**Background:**

We investigated whether a short, 5-min magnetic resonance imaging (MRI) protocol consisting of only axial T2-weighted and diffusion-weighted imaging (DWI) sequences can discriminate between tonsillar infections, peritonsillar abscesses and deeply extending abscesses in a retrospective, blinded, multireader setting.

**Methods:**

We included patients sent by emergency physicians with suspected pharyngotonsillar infections who underwent emergency neck 3-T MRI from April 1 2013 to December 31 2018. Three radiologists (with 10−16 years of experience) reviewed the images for abscesses and their extension into deep neck spaces. Data were reviewed first using only axial T2-weighted Dixon images and DWI (short protocol) and second including other sequences and contrast-enhanced T1-weighted Dixon images (full protocol). Diagnostic accuracy, interobserver agreement, and reader confidence were measured. Surgical findings and clinical course served as standard of reference.

**Results:**

The final sample consisted of 52 patients: 13 acute tonsillitis with no abscesses, 19 peritonsillar abscesses, and 20 deeply extending abscesses. Using the short protocol, diagnostic accuracy for abscesses across all readers was good-to-excellent: sensitivity 0.93 (95% confidence interval 0.87−0.97), specificity 0.85 (0.70−0.93), accuracy 0.91 (0.85−0.95). Using the full protocol, respective values were 0.98 (0.93−1.00), 0.85 (0.70−0.93), and 0.95 (0.90−0.97), not significantly different compared with the short protocol. Similar trends were seen with detecting deep extension. Interobserver agreement was similar between protocols. However, readers had higher confidence in diagnosing abscesses using the full protocol.

**Conclusions:**

Short MRI protocol showed good-to-excellent accuracy for tonsillar abscesses. Contrast-enhanced images improved reader confidence but did not affect diagnostic accuracy or interobserver agreement.

**Relevance statement:**

Short protocol consisting only of T2-weighted Dixon and DWI sequences can accurately image tonsillar abscesses, which may improve feasibility of emergency neck MRI.

**Key points:**

• The short 3-T MRI protocol (T2-weighted images and DWI) was faster (5 min) than the full protocol including T1-weighted contrast-enhanced images (24 min).

• The short 3-T MRI protocol showed good diagnostic accuracy for pharyngotonsillar abscesses.

• Contrast-enhanced sequences improved reader confidence but did not impact diagnostic accuracy or interobserver agreement.

**Graphical Abstract:**

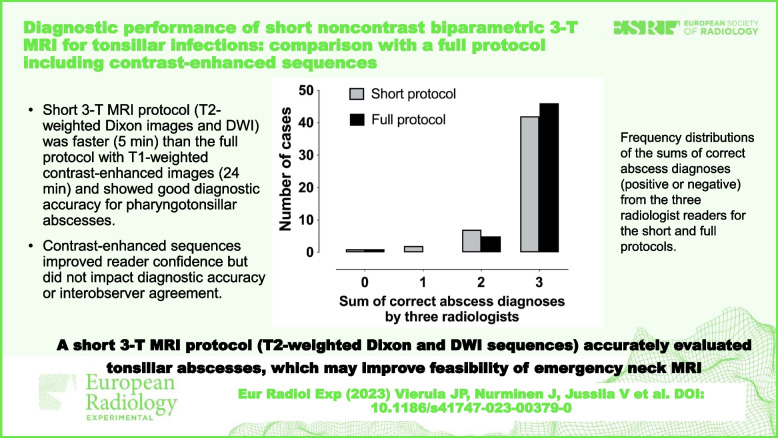

## Background

Tonsillar infections are common [1]. Most of these are mild and can be managed conservatively without diagnostic imaging or surgical interventions. However, tonsillar infections can lead to a peritonsillar abscess (PTA), defined by pus accumulating in the space between the tonsillar capsule and the superior pharyngeal constrictor muscle. From there, a PTA may further spread into parapharyngeal or retropharyngeal spaces by breaching the muscle wall, thus becoming a deep neck infection. These deep neck infections are potentially life-threatening conditions requiring prompt surgical drainage and other interventions [2]. A simple PTA is often diagnosed clinically without any emergency imaging. Imaging may be required after unsuccessful drainage attempts or in clinical suspicion of a deeply extending abscess. Imaging aims to verify the correct diagnosis of tonsillar infection, detect or exclude abscess and its potential deep extensions, and identify other potential complications [3].

In clinical practice, contrast-enhanced computed tomography (CECT) is often used in acute neck infections [4] but it has limited soft tissue contrast to separate drainable abscesses from surrounding phlegmon [5]. Magnetic resonance imaging (MRI) has recently emerged as an alternative imaging method with higher diagnostic accuracy than CECT [6]. Specifically, MRI is highly accurate in differentiating between simple PTAs and deeply extending abscesses in patients with acute pharyngotonsillar infections [7]. Abscesses appear on MRI as non-enhancing collections, hyperintense or isointense on T2-weighted images, with central restricted diffusion, manifested as low apparent diffusion coefficient (ADC) values on diffusion-weighted imaging (DWI) [7]. However, neck MRI is limited by long scan times (usually about 30 min), which is disadvantageous because patients with pharyngotonsillar infections may have difficulty lying supine due to throat swelling. In addition, MRI protocols often include intravenous administration of gadolinium-based contrast agents, which require intravenous access, increase cost, and may infrequently cause adverse effects. Therefore, a shorter unenhanced MRI protocol would be beneficial and likely improve the generalizability of MRI in acute neck imaging.

The primary aim of this study is to examine whether a short, 5-min noncontrast MRI protocol, consisting of only T2-weighted and DWI sequences can reliably discriminate between tonsillar infections, PTAs, and deeply extending abscesses in a retrospective, blinded, multireader comparison. The secondary aim was to evaluate whether the full protocol, including contrast-enhanced images, significantly increases diagnostic accuracy. We chose to investigate the spectrum of pharyngotonsillar infections because they represent the most common type of acute neck infections, and previous work indicates that abscesses might be discriminated from adjacent tonsillar tissue using T2-weighted and DWI sequences [7].

## Methods

For this retrospective cohort study in a tertiary hospital that primarily uses MRI for neck emergencies, data have been previously published for patient selection, MRI and its interpretation, and extraction of medical and surgical information [6, 7]. Briefly, we obtained study permission from the hospital district board for this retrospective cohort study. The requirement of patient consent was waived due to the retrospective nature of the study.

The inclusion criteria were as follows: (1) emergency MRI between April 1, 2013, and December 31, 2018, for suspected neck infection; (2) MRI evidence of infection, *i.e.*, high signal on fat-suppressed T2-weighted Dixon images suggesting edema or high signal on fat-suppressed contrast-enhanced T1-weighted Dixon images suggesting abnormal tissue enhancement; (3) a final clinical diagnosis of a tonsillar infection; and (4) diagnostic image quality as determined by the radiologist reading the study. The exclusion criterion was lack of clinical and surgical data.

**Fig. 1:**
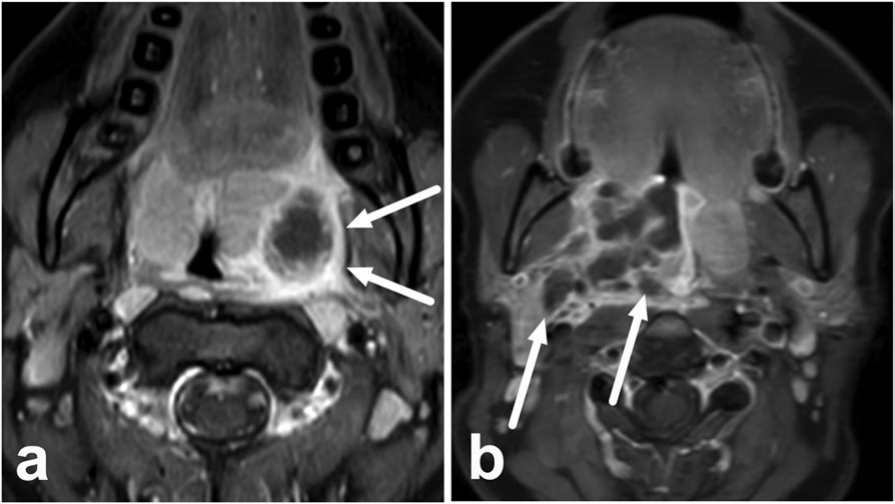
Examples of a simple peritonsillar abscess (**a**) and a deeply extending abscess (**b**) on axial fat-suppressed postcontrast T1-weighted Dixon images in different patients. Abscesses present as areas of non-enhancement in these images. In the case of a peritonsillar abscess, the abscess is contained within the enhancing superior constrictor muscles (arrows, **a**). In the deeply extending abscess, non-enhancing abscess material (arrows) can be seen extending into the parapharyngeal space, far lateral from the muscle border (**b**)

**Fig. 2:**
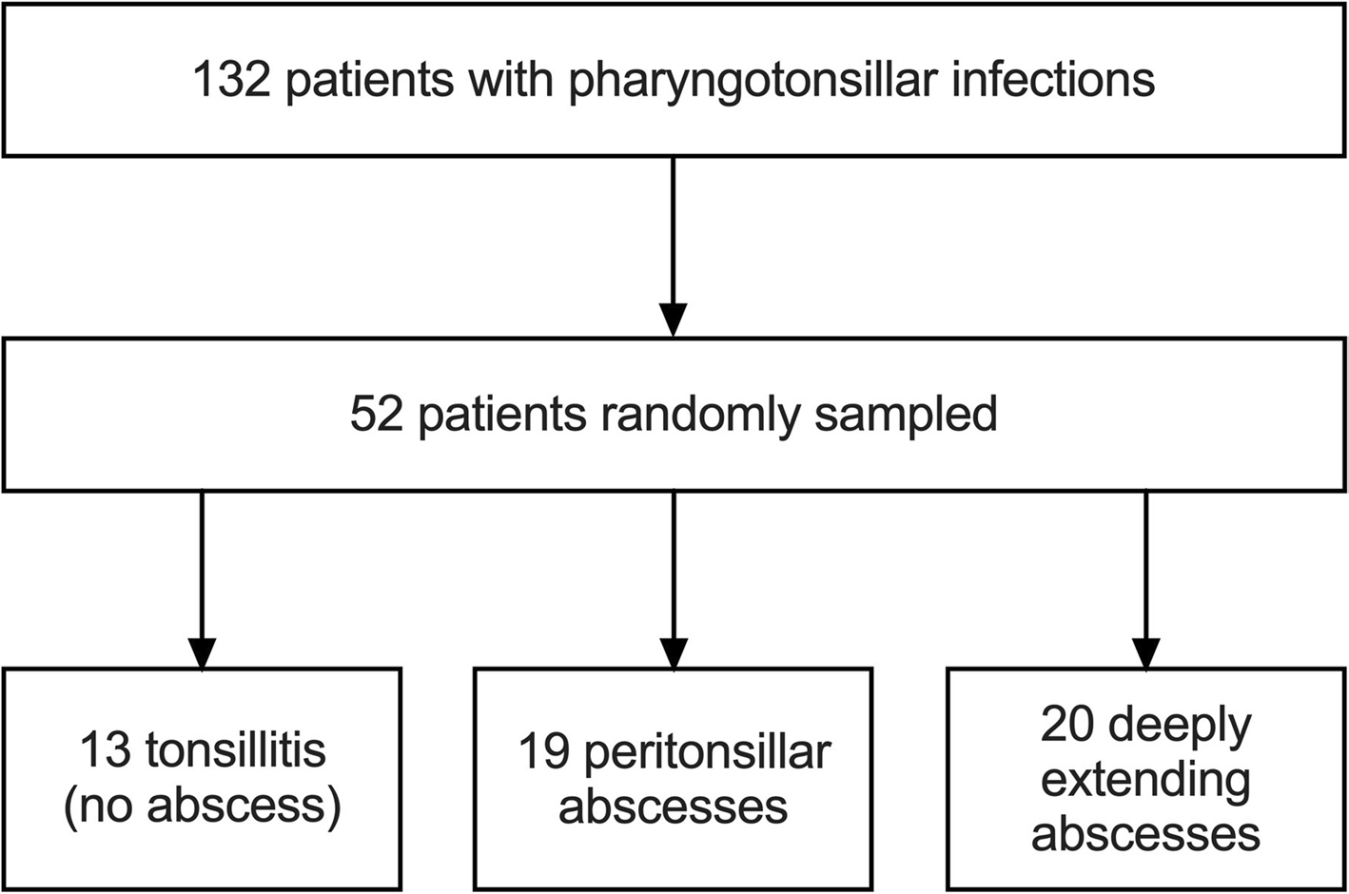
Flowchart of the patient population

Patients underwent emergency neck MRI with the Ingenia 3-T system (Philips Healthcare, Best, The Netherlands) using a dS HeadNeckSpine coil configuration and a gadolinium-based intravenous contrast agent (Dotarem®; Guerbet, Villepinte, France). Data from medical records, including surgical data, were used to categorize cases into mere tonsillar infections, PTAs, or deeply extending abscesses (Fig. 1). These cases were randomly sampled within each class from a previously published larger cohort of consecutive patients [7] (Fig. 2). Because tonsillitis without abscesses is rare on imaging, these cases were intentionally overrepresented in our sample, and then cases with abscesses were included by up to 1.5 times the number of cases with tonsillitis to avoid a severe imbalance of classes. Cases were randomly sampled from within the three classes according to this scheme. Surgical findings were used as a reference standard for abscesses. For mere tonsillar infections without abscesses, a true negative was considered on clinical grounds when the patient recovered uneventfully without surgery after a negative MRI. We used ratios between the suspected abscess and the tonsil calculated from apparent diffusion coefficient (ADC) and the signal intensity on T2-weighted images (T2-SI), as previously described [7].

For each patient, we retrospectively curated two sets of MRI data: a short biparametric protocol including only axial T2-weighted Dixon images and DWI; and a full protocol including, in addition to the short protocol, also coronal T2-weighted Dixon, axial T1-weighted, and axial, coronal, and sagittal postcontrast T1-weighted Dixon images (Table 1).

**Table 1:** Sequences included in the short and the full protocols

Sequence	Short protocol	Full protocol	Parameters	Scan time
T2 Dixon axial	Yes	Yes	Slice thickness 4 mm, TE 100 ms, TR 3,021 ms, flip angle 90°	3:46
DWI axial	Yes	Yes	Slice thickness 4 mm, TE 87 ms, TR 3,981 ms, *b* value 1,000 s/mm^2^, flip angle 90°	0:48
T2 Dixon coronal	No	Yes	Slice thickness 3.5 mm, TE 80 ms, TR 3,210 ms, flip angle 90°	5:14
T1 TSE axial	No	Yes	Slice thickness 4 mm, TE 10 ms, TR 641 ms, flip angle 90°	4:24
T1 Dixon axial postcontrast	No	Yes	Slice thickness 4 mm, TE 7 ms, TR 634 ms, flip angle 90°	3:29
T1 Dixon coronal postcontrast	No	Yes	Slice thickness 3.5 mm, TE 14 ms, TR 560 ms, flip angle 90°	3:08
T1 Dixon sagittal postcontrast	No	Yes	Slice thickness 3 mm, TE 14 ms, TR 630 ms, flip angle 90°	3:06
Total scan time (min:s)	4:34	23:55		

*DWI* Diffusion-weighted imaging, *TE* Echo time, *TR* Repetition time, *TSE* Turbo spin echo

In the multireader study, three board-certified radiologists (with 16, 11, and 10 years of experience) independently and retrospectively analyzed the images, blinded to all medical and surgical information. Cases were evaluated for any abscesses (yes/no), and, further, deeply extending abscesses to parapharyngeal and/or retropharyngeal spaces (yes/no). Reader confidence in this decision-making was assessed with a 5-step grading system where step 1 represented “very high uncertainty”, step 2 “moderate uncertainty”, step 3 “borderline case”, step 4 “moderate confidence”, and step 5 “very high confidence” in the decision. Images were reviewed in two separate sessions: first, with the short protocol, and second, with the full protocol (Table 1). Reading sessions were at least four weeks apart, and cases were reorganized between sessions to avoid recall bias. Guidelines for image interpretation were made available to the readers well before the sessions in the form of our previous publication on MRI findings in acute tonsillar infections [7].

Size of abscesses was measured as the maximal diameter of the non-enhancing collection [7]. ADC and T2-SI of abscesses and tonsils were measured as mean values from single regions of interest manually delineated on representative axial slices.

Reader assessments were compared with those from surgical data and patient records. Sensitivity, specificity, accuracy, positive and negative predictive values were calculated for PTAs and deeply extending abscesses as defined surgically, as pooled results from all three readers. Differences in sensitivity and specificity between the short and the full protocols were assessed using binomial tests. Interrater reliability was estimated using κ values [8]. Changes in confidence scores were tested with a paired samples *t*-test. Statistical data were analyzed with IBM SPSS Statistics for Mac (version 26, copyright IBM Corporation 2019): *p* values lower than 0.05 were considered statistically significant.

## Results

The final study sample consisted of 13 acute tonsillitis with no abscesses, 19 PTAs, and 20 deeply extending abscesses, adding up to 52 patients (Table 2). No intratonsillar abscesses were detected or included.

**Table 2:** Demographic, clinical, and radiological data from the study subjects

	Tonsillitis	Peritonsillar abscess	Abscess deep extension	Total	*p* value
Number	13	19	20	52	–
Age (years)	39 ± 14	40 ± 21	47 ± 17	43 ± 18	0.347
Female (%)	0%	37%	55%	35%	0.005
Symptom duration (days)	4.9 ± 2.3	5.5 ± 3.9	4 ± 4	4.8 ± 3.6	0.422
C-reactive protein (mg/mL)	169 ± 67	105 ± 106	192 ± 97	155 ± 100	0.018
White blood cell count	13 ± 6	13 ± 4	16 ± 6	14 ± 5	0.133
Abscess largest diameter (mm)	0	22 ± 10	54 ± 33	29 ± 30	< 0.001

Data for continuous variables are given as mean ± standard deviation

**Fig. 3:**
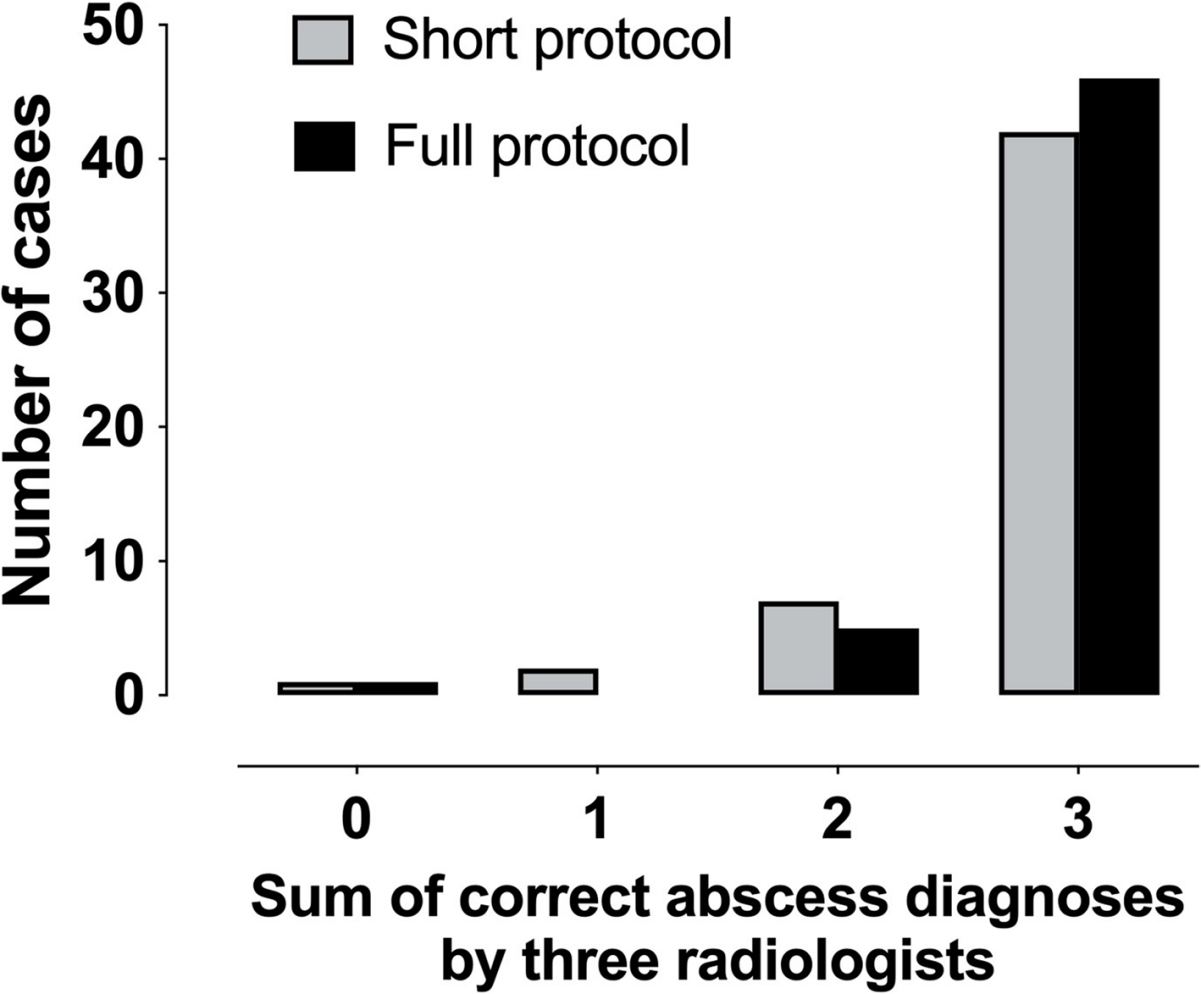
Frequency distributions of the sums of correct abscess diagnoses (positive or negative) from the three radiologist readers for the short and full protocols

Across all readers, the short biparametric MRI protocol had a sensitivity, specificity, and accuracy of 0.93, 0.85, and 0.91 for any abscesses, respectively (Table 3). Corresponding values for the full contrast-enhanced protocol were 0.98, 0.85, and 0.95, respectively (Table 3). Change from short to full protocol did not reach statistical significance for either sensitivity (*p* = 0.109) or specificity (*p* = 1.000). The minor impact of the full contrast-enhanced protocol was also evident from the small change in the sum of correct abscess diagnoses across readers (Fig. 3).

**Table 3:** Multireader diagnostic performance, interobserver agreement, and reader confidence for short biparametric and full MRI protocols for detecting abscesses and deep extension

	Short biparametric protocol		Full protocol including contrast-enhanced sequences		*p* value (short *versus* full protocol)	
	Abscess	Deep extension	Abscess	Deep extension	Abscess	Deep extension
Sensitivity	0.93 [0.87–0.97]	0.73 [0.61–0.83]	0.98 [0.93–1.00]	0.70 [0.57–0.80]	0.109	0.754
Specificity	0.85 [0.70–0.93]	0.92 [0.84–0.96]	0.85 [0.70–0.93]	0.97 [0.91–0.99]	1.000	0.227
Positive predictive value	0.95 [0.89–0.98]	0.85 [0.72–0.92]	0.95 [0.89–0.98]	0.93 [0.81–0.98]		
Negative predictive value	0.80 [0.66–0.90]	0.85 [0.76–0.99]	0.94 [0.80–0.99]	0.84 [0.76–0.90]		
Accuracy	0.91 [0.85–0.95]	0.85 [0.78–0.89]	0.95 [0.90–0.97]	0.87 [0.80–0.91]		
Interobserver κ	0.70 [0.55–0.86]	0.60 [0.45–0.75]	0.82 [0.66–0.97]	0.66 [0.51–0.81]	0.312	0.929
Confidence	4.3 [0.96]		4.6 [0.72]		< 0.001	

Values in square brackets represent the upper and lower bounds of the 95% confidence interval for diagnostic accuracy metrics and κ, while they represent standard deviation for confidence

Regarding deep extension of abscesses, the short biparametric MRI protocol had a sensitivity, specificity, and accuracy of 0.73, 0.92, and 0.85, respectively, and the full contrast-enhanced protocol had corresponding numbers of 0.70, 0.97, and 0.87 (Table 3). Similar to the detection of any abscesses, change in neither sensitivity (*p* = 0.754) nor specificity (*p* = 0.227) for deep extension reached statistical significance.

**Fig. 4:**
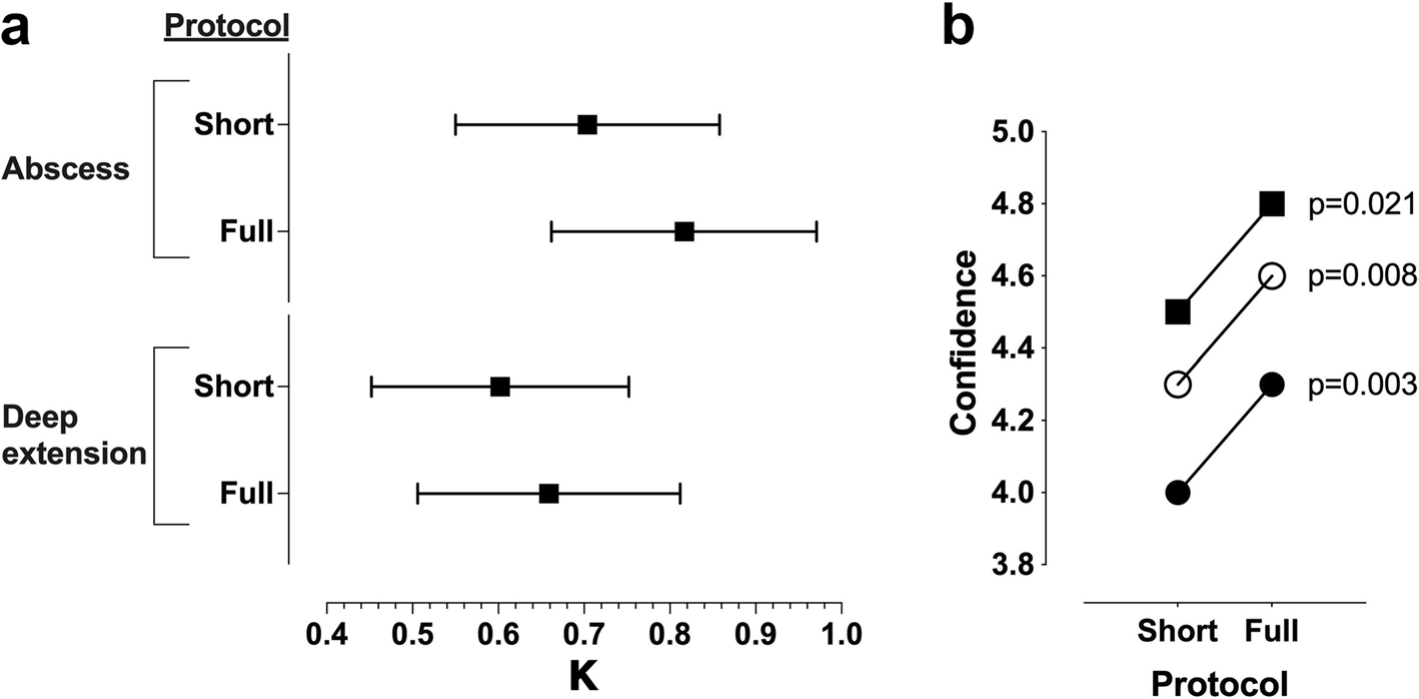
Interobserver agreement (κ) values for the short and full protocols for detecting abscesses and their deep extension (**a**) and confidence estimates for the three radiologist readers separately (open circle, black circle, and black square) while reviewing the short and full protocols (**b**)

Interobserver κ for the detection of any abscesses was 0.70 for the short protocol and 0.82 for the full protocol (*p* = 0.312). Corresponding numbers for the detection of deep extension were 0.60 and 0.66, respectively (*p* = 0.929). These results indicate moderate to substantial interobserver agreement even with the short protocol, and no statistically significant change between short and full protocols (Fig. 4). Reader confidence in abscess diagnostics, on a scale of 1-to-5, improved significantly in all readers from the short protocol (average 4.3) to the full protocol (average 4.6) (*p* = 0.003–0.021 for individual readers, *p* < 0.001 for data from all readers) (Fig. 4).

Among the 39 patients with surgically proven abscesses, in only five patients (15%) one or more of the readers did not detect an abscess with the short protocol (false negatives). Compared with those in whom an abscess was detected (true positives), they were smaller in diameter (15 *versus* 42 mm, *p* = 0.055) and had a higher ADC ratio (0.80 *versus* 0.61, *p* = 0.055), both at trend-level significance, but similar T2-SI ratio (1.28 *versus* 1.36, *p* = 0.527). Finally, the radiologists reported lower average confidence for false negatives (3.7) than for true positives (4.4) when reviewing the short protocol (*p* = 0.009) but not when examining the full protocol (4.5 *versus* 4.8, *p* = 0.330).

**Fig. 5:**
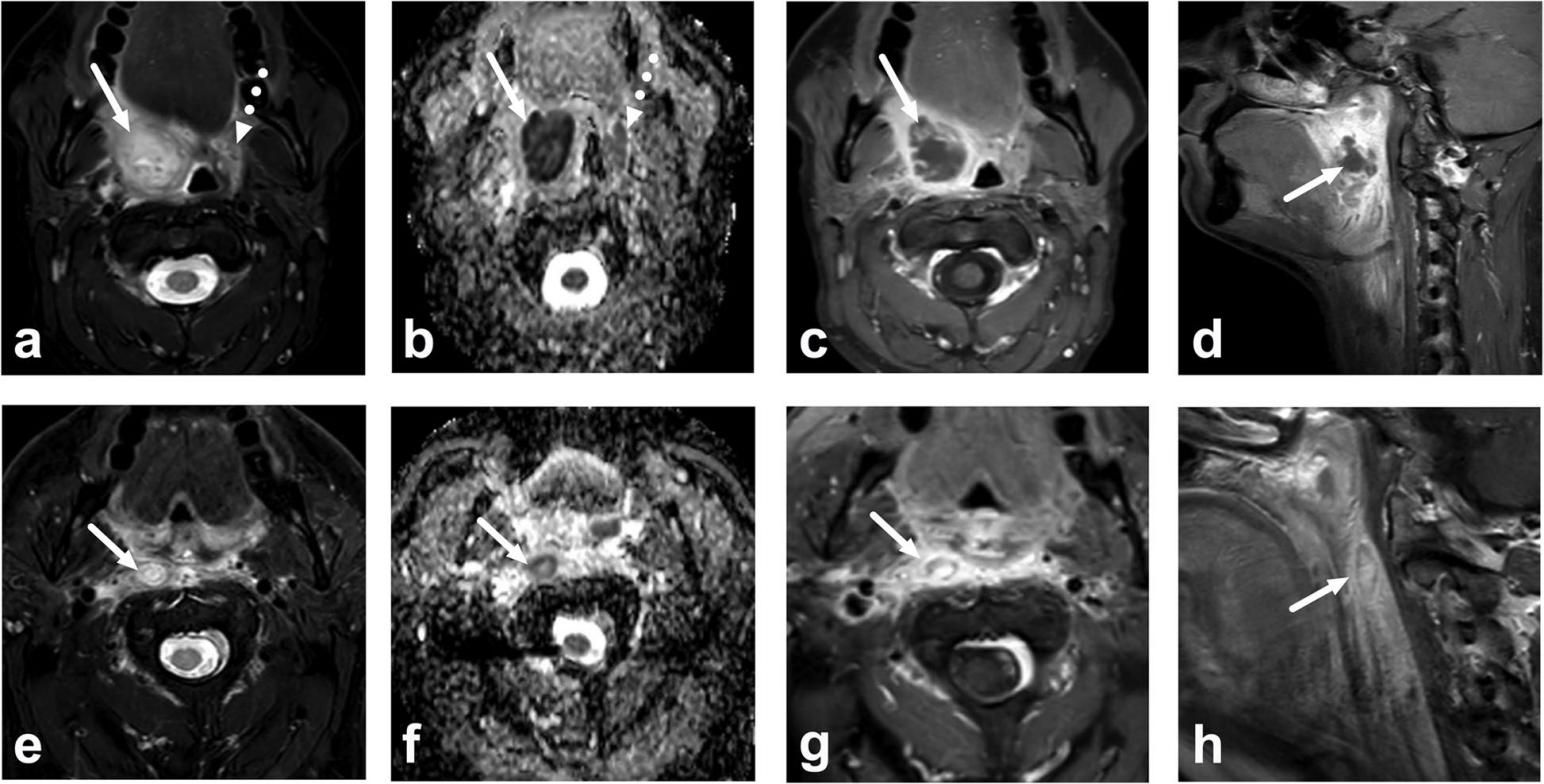
Examples of a true positive (**a**–**d**) and a false positive (**e**–**h**) peritonsillar abscess (PTA) interpreted using the short protocol. Images are axial fat-suppressed T2-weighted images (**a** and **e**), apparent diffusion coefficient (ADC) maps (**b** and **f**), and axial (**c** and **g**) and sagittal (**d** and **h**) postcontrast T1-weighted images. True positive PTA, top row (**a**–**d**): a T2-hyperintense collection with low ADC (arrows) can easily be separated from normal tonsillar tissue (dotted arrow). All three radiologists correctly identified the PTA using the short protocol (**a** and **b**) and subsequently using contrast-enhanced images (**c** and **d**). False positive case, bottom row (**e**–**h**): a small T2-hyperintense (**e**) area with low ADC (**f**) can be seen in the retropharyngeal space (arrows), interpreted as a PTA by one of three radiologists using the short protocol. However, postcontrast images (**g** and **h**) clearly demonstrate contrast enhancement in this ovoid lesion (arrows), thus ruling out an abscess and suggesting a retropharyngeal lymph node

**Fig. 6:**
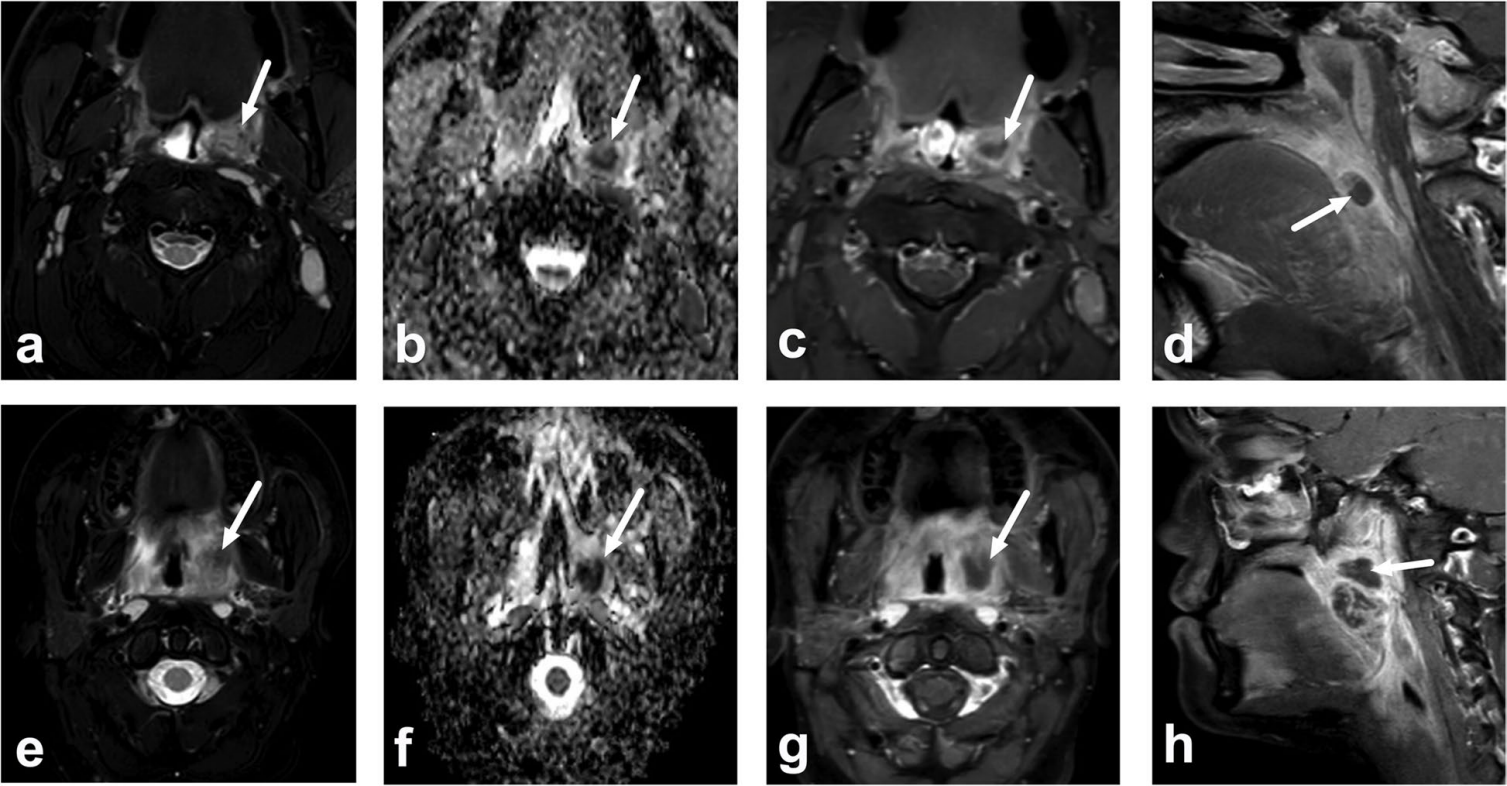
Examples of two difficult peritonsillar abscess (PTA) cases for the short protocol (**a**–**d** and **e**–**h**). Images are axial fat-suppressed T2-weighted images (**a** and **e**), apparent diffusion coefficient (ADC) maps (**b** and **f**), axial fat-suppressed postcontrast T1-weighted images (**c** and **g**), and sagittal postcontrast T1-weighted images (**d** and **h**). Both cases demonstrate areas of intermediate to low T2-signal (**a** and **e**) and low ADC (**b** and **f**) (arrows) in the region of the left palatine tonsil. Using the short protocol, one of three radiologists failed to identify the PTA in the first (top row), and all three radiologists missed the PTA in the second patient (bottom row), although the non-enhancing abscess (arrows in **c**, **d**, **g**, and **h**) was clearly identified using the full protocol by all readers. In both cases, abscesses is not well seen on T2-weighted imaging because of intermediate to low T2-signal, and low ADC can be mistaken for normal tonsillar tissue in patients with small palatine tonsils

Examples of typical or challenging cases are presented in Figs. 5 and 6.

## Discussion

We examined the diagnostic accuracy of a short (5-min) biparametric noncontrast MRI protocol for tonsillar abscesses, in comparison with full contrast-enhanced protocol, in a retrospective, blinded, multireader setting. The short axial protocol showed a good-to-excellent diagnostic accuracy for tonsillar abscesses, and only a few small abscesses were missed. Adding other images, including those obtained with contrast-enhanced sequences, numerically improved sensitivity for any abscesses, specificity for deep extension, and interobserver agreement, without reaching statistical significance. Contrast-enhanced sequences slightly improved radiologist confidence. These results suggest that a shorter and possibly better tolerated and cheaper MRI protocol may adequately answer clinically relevant questions in patients with acute pharyngotonsillar infections.

In emergency imaging, high sensitivity is often desired to avoid false negatives and potential delays in appropriate treatment. In the current study, sensitivity did not statistically significantly change from short to full protocol. With this issue in mind, what abscesses were missed with the short protocol? Compared with true positives, these few false negative abscesses were smaller and had ADC values higher than those of the palatine tonsils. High ADC likely makes them challenging to discriminate from the adjacent tonsils because ADC is usually lower in abscesses than in the tonsils [7]. Thus, the short protocol with only T2-weighted Dixon and DWI may miss small abscesses with relatively high ADC. These patients could likely be managed with short-term follow-up, with the caveat of a potential delay in initiating appropriate treatment.

The short protocol lasts only about 5 min, which makes it practically as fast as CECT in the emergency department. Even this short MRI protocol yielded higher diagnostic accuracy performance than previous studies using CECT [5]. In fact, there is limited information on the diagnostic accuracy of CECT for surgically confirmed simple PTAs. A large CECT study with surgical confirmation found a positive predictive value of 0.80 for parapharyngeal abscesses [9], which is considerably lower than the 0.95 found in the current study with the short MRI protocol. In fact, a positive predictive value of 0.95 is as high as that previously reported in our validation cohort using the full contrast-enhanced protocol across multiple types of neck infections [6]. Because of partial verification bias (patients with abscesses on imaging are much more likely to undergo surgical confirmation than patients without abscesses), positive predictive value is often a more reliable metric to compare modalities than sensitivity and specificity [5].

In many clinical MRI indications, using contrast-enhanced sequences is justified. In general, contrast agent improves the delineation of non-enhancing fluid collections suspicious of abscesses in acute neck infection [10]. The use of contrast-agent possesses, however, some potential disadvantages. Scan times are longer, and precontrast T1-weighted images are usually acquired for baseline assessment, although they probably have limited value in pharyngotonsillar infections. Using a contrast agent also requires intravenous access, a minor but potentially time-consuming invasive medical procedure. Further, although gadolinium-based contrast agents are generally safe and well tolerated, their use in some patients, such as those with kidney failure, may not be justified. Many open questions exist about the long-term health effects of gadolinium retained from contrast agents [11].

Examples of typical and challenging study cases are given in Figs. 5 and 6. In the short MRI protocol, the T2-weighted axial Dixon sequence shows soft tissue edema and high signal intensity associated with fluid in a potential abscess, and the DWI sequence is used to determine potential diffusion restriction associated with the purulence of the collection (abscess). The interpretation may, however, be complicated if the T2-SI ratio between the abscess and the tonsil is low (Fig. 6). Whether previous drainage attempts change the T2-SI or ADC of the collection has yet to be discovered. In addition, a lymph node can have restricted diffusion due to high cellularity. Therefore, a focal intermediate T2-SI lesion with restricted diffusion can denote a lymph node and not an abscess (Fig. 5), suggesting that the diagnosis of suppurative lymphadenitis requires a contrast agent. However, the diagnosis of suppurative lymphadenitis can be challenging even with a contrast agent [10, 12].

The strengths of our study include high-quality 3-T MRI from patient cohort surgically (or clinically) validated and a blinded multireader setting. Our study is limited by the relatively small sample size per patient group, which is largely driven by the small number of patients with tonsillitis only (no abscess), which is a rare MRI finding [7]. So, we could not perform a formal noninferiority study with predetermined margin of noninferiority and the lack of significance in some comparisons could be due to lack of statistical power because of a small sample size. We included only patients with pharyngotonsillar infections because they represent the most common type of acute neck infection in our clinical practice [6]. Therefore, we do not know if the short protocol would perform equally well in other types of infections, such as odontogenic neck infections [13]. Finally, the short protocol is heavily dependent on the technical success of the DWI scan, which may be distorted in the neck area by artifacts from patient movement or foreign metal objects (such as dental hardware). No patients in this cohort were excluded because of motion artifacts.

Our short “virtual” MRI protocol consisted of only T2-weighted Dixon sequences and DWI sequences in the axial plane. The full MRI protocol added not only contrast-enhanced sequences in all three planes but also unenhanced coronal T2-weighted Dixon images and unenhanced axial T1-weighted images. Thus, as a further limitation of this study, we cannot determine to what extent the subtle, statistically non-significant improvements in diagnostic accuracy using the full protocol were due to contrast-enhanced T1-weighted or the other additional images. Based on clinical experience, we suspect that contrast-enhanced images provided the most useful additional information and confidence.

Shortening the duration of MRI protocols is an attractive strategy in diagnostic radiology to reduce economic burden, save time, and improve the availability of MRI. Although previous studies on neck infections are lacking, this approach has proven successful in other fields of radiology, such as prostate [14], abdominal [15], and breast imaging [16]. For example, similar diagnostic accuracy to detect prostate cancer at a lower cost has been proven for an abbreviated prostate MRI protocol [17]. Faster MRI acquisition methods based on deep learning will likely accelerate this development [18].

In conclusion, a short 5-min 3-T MRI protocol consisting only of T2-weighted Dixon and DWI sequences can accurately identify and characterize pharyngotonsillar abscesses and seems to perform better than conventional CECT in previous studies. When properly validated, short MRI protocols may pave the way for more widespread use of this modality in emergencies.

## Data Availability

Patient data cannot be publicly shared because of the national legislature on patient data.

## Abbreviations

ADC: Apparent diffusion coefficient
CECT: Contrast-enhanced computed tomography
DWI: Diffusion-weighted imaging
MRI: Magnetic resonance imaging
PTA: Peritonsillar abscess
T2-SI: Signal intensity on T2-weighted images
